# Shed syndecan-2 inhibits angiogenesis

**DOI:** 10.1242/jcs.153015

**Published:** 2014-11-01

**Authors:** Giulia De Rossi, Alun R. Evans, Emma Kay, Abigail Woodfin, Tristan R. McKay, Sussan Nourshargh, James R. Whiteford

**Affiliations:** 1 William Harvey Research Institute, Barts and The London School of Medicine and Dentistry, Queen Mary University of London, Charterhouse Square, London EC1M 6BQ, UK; 2 Division of Biomedical Sciences, St. George's University of London, Cranmer Terrace, London SW17 0NE, UK

**Keywords:** Angiogenesis, Inflammation, Syndecan, Integrin, Endothelial cell migration

## Abstract

Angiogenesis is essential for the development of a normal vasculature, tissue repair and reproduction, and also has roles in the progression of diseases such as cancer and rheumatoid arthritis. The heparan sulphate proteoglycan syndecan-2 is expressed on mesenchymal cells in the vasculature and, like the other members of its family, can be shed from the cell surface resulting in the release of its extracellular core protein. The purpose of this study was to establish whether shed syndecan-2 affects angiogenesis. We demonstrate that shed syndecan-2 regulates angiogenesis by inhibiting endothelial cell migration in human and rodent models and, as a result, reduces tumour growth. Furthermore, our findings show that these effects are mediated by the protein tyrosine phosphatase receptor CD148 (also known as PTPRJ) and this interaction corresponds with a decrease in active β1 integrin. Collectively, these data demonstrate an unexplored pathway for the regulation of new blood vessel formation and identify syndecan-2 as a therapeutic target in pathologies characterised by angiogenesis.

## INTRODUCTION

Angiogenesis is a feature of development, wound healing, tumorigenesis and chronic inflammatory conditions, such as rheumatoid arthritis and atherosclerosis (Carmeliet, 2003; Mapp and Walsh, 2012). The process requires the transition of endothelial cells from a quiescent state to a migratory and proliferative phenotype, and is stimulated and controlled by growth factors, most notably vascular endothelial growth factor (VEGF) (Sakurai and Kudo, 2011). Endothelial cell migration is a crucial component of angiogenesis and integrins are the major drivers of this response (Ramjaun and Hodivala-Dilke, 2009). Integrins exist in various activation states on the cell surface and this modulates the migratory and adhesive characteristics of cells through interactions with the extracellular matrix (ECM). Other cell surface receptors also interact with ECM ligands leading to signalling cascades that can alter the activation state of integrins (Streuli and Akhtar, 2009). The four-member syndecan family of heparan sulphate proteoglycans (HSPGs) are an example of such molecules.

The syndecans are a family of transmembrane receptors with roles in cell adhesion, migration and growth factor signalling. In common with the other family members, syndecan-2 has a short cytoplasmic domain, a single-pass transmembrane domain and a larger extracellular domain, which is modified toward the N-terminus with heparan sulphate (HS) side chains and can be shed from the cell surface. Many cell types shed syndecan ectodomains through the action of matrix metalloproteinases (MMPs) in response to a variety of stimuli including inflammatory mediators, but the biological implications of this response are not fully understood (Manon-Jensen et al., 2010). In the present study, the possibility that shed syndecan-2 might be a regulator of angiogenesis was investigated.

There is a considerable body of work to suggest that syndecan extracellular core proteins have domains that influence cell behaviour. For example, cell adhesion regulatory domains have been identified in all four syndecan family members (De Rossi and Whiteford, 2013a; De Rossi and Whiteford, 2013b; McFall and Rapraeger, 1997; McFall and Rapraeger, 1998; Whiteford et al., 2007; Whiteford and Couchman, 2006). How and when these regulatory sequences function and their relevance to biological processes has yet to be determined. Previously, we have shown that the syndecan-2 extracellular core protein (S2ED) can support fibroblast attachment and spreading (Whiteford et al., 2007). Here, we show that S2ED shed from cells acts to inhibit angiogenesis in both rodent and human models through a paracrine interaction with the protein tyrosine phosphatase receptor CD148. This interaction leads to a de-activation of β1 integrins resulting in a reduction in endothelial cell migration.

## RESULTS

### Constitutively released syndecan-2 ectodomain inhibits angiogenesis in a xenograft tumour model

**Fig. 1.**
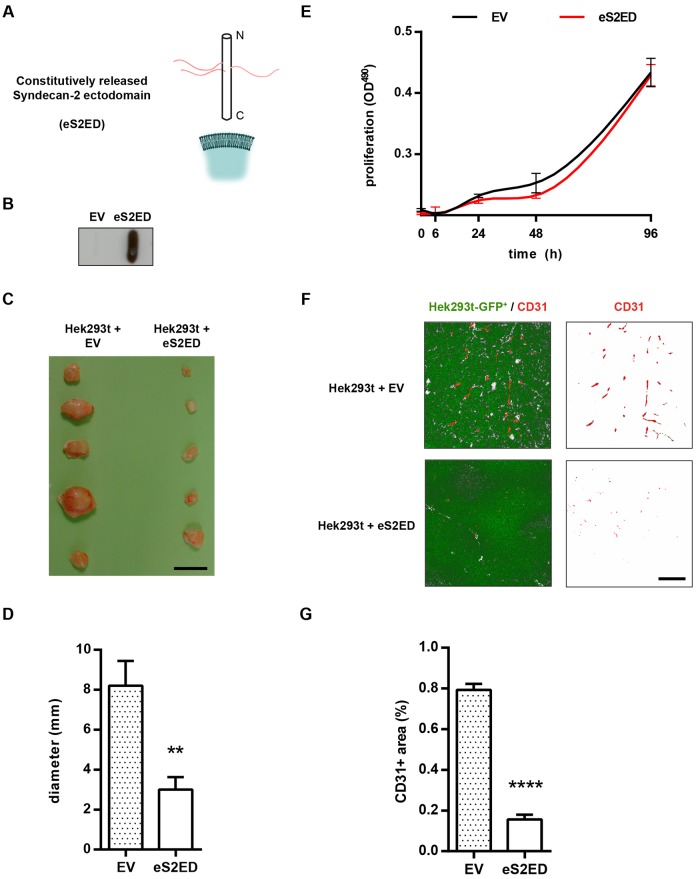
**Constitutively released syndecan-2 extracellular core protein from HEK293t cells inhibits angiogenesis in a xenograft tumour model.** (A) Schematic diagram of syndecan-2 lacking both cytoplasmic and transmembrane domains (eS2ED), which is constitutively released from HEK293t cells. (B) Dot blot of conditioned media from cells transfected with either empty vector (EV) or eS2ED constructs. Dot blots were probed with the anti-HA antibody, and a strong signal is obtained from cells transfected with eS2ED. (C) Constitutively released syndecan-2 inhibits tumour growth. Micrograph of tumours from SCID-SHO mice injected with either empty vector cells or eS2ED cells. Scale bar: 1 cm. (D) Tumours derived from eS2ED cells have smaller diameter. Error bars represent the s.e.m. **P<0.01 (*n* = 5 tumours per condition, Student's *t*-test. (E) Empty vector cells and S2ED cells have the same growth kinetics over 96 h in culture. (F) Immunofluorescence staining of tumour sections derived from empty vector cells and eS2ED-transfected cells for the endothelial cell marker CD31 (red; tumour cells are GFP positive). Considerably more CD31-positive structures are evident in tumours derived from cells transfected with empty vector as compared the tumours in which syndecan-2 is constitutively released. Scale bar: 200 µm. (G) Quantification of the area of micrographs positive for CD31 was performed and expressed as a percentage of the GFP-positive tumour cells. Data are the mean from three separate images from sections of three tumours per group. Error bars represent the s.e.m. ****P<0.0001 (unpaired Student's *t*-test).

To investigate the potential effects of shed syndecan-2 on angiogenesis we established a mammalian expression system in which a constitutively released form of the molecule (lacking both transmembrane and cytoplasmic domains, M^1^–F^141^, eS2ED) was expressed in HEK293t cells (Fig. 1A). The HA epitope was also incorporated into eS2ED between D^27^ and K^28^ enabling us to readily detect eS2ED in conditioned media as compared to controls transfected with empty vector (Fig. 1B; supplementary material Fig. S1). Using these cell lines, we investigated the anti-angiogenic properties of shed syndecan-2 in a xenograft tumour model. Specifically, SCID-SHO mice were injected with empty vector cells or cells that constitutively released syndecan-2 (eS2ED) and tumour sizes were quantified at 21 days. Mice injected with control (empty vector) cells developed vascularised tumours that were significantly larger both in terms of diameter and weight as compared to tumours generated in mice injected with the eS2ED cells (Fig. 1C,D; supplementary material Fig. S2). We compared the growth kinetics in culture between empty vector cells and eS2ED-transfected cell lines and found no differences, indicating that cell proliferation defects were not the reason tumours derived from eS2ED cells failed to develop (Fig. 1E). Immunofluorescence staining of tumour sections for the endothelial cell marker CD31 (also known as PECAM1) revealed that tumours derived from empty vector control cells contained CD31-positive structures consistent with blood vessels. CD31-positive cells were greatly reduced in tumour sections derived from cells constitutively releasing syndecan-2 (Fig. 1F,G). These findings suggest that constitutive release of the syndecan-2 ectodomain in this model is inhibitory to angiogenesis.

### The shed syndecan-2 ectodomain inhibits angiogenesis

**Fig. 2.**
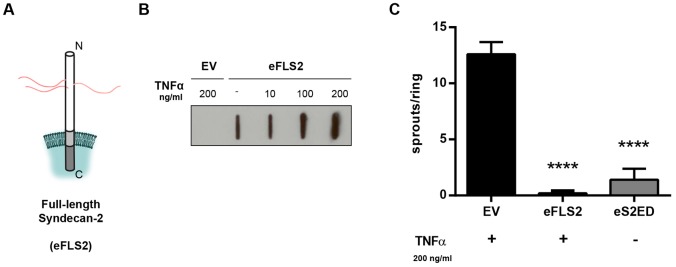
**Shed syndecan-2 from HEK293t cells has anti-angiogenic properties.** (A) Schematic diagram of full-length HA-tagged syndecan-2 (eFLS2), which was expressed in HEK293t cells. (B) Syndecan-2 is shed from HEK293t cells after stimulation with TNFα in a dose-dependant manner. eFLS2 cells were incubated for 4 h in serum-free media with the indicated concentration of TNFα, and conditioned media were assayed by dot blot using the anti-HA antibody. EV, empty vector. (C) Shed syndecan-2 is anti-angiogenic. Empty vector cells and eFLS2 cells were treated with TNFα (200 ng/ml) for 4 h and the shed proteoglycans were purified by anion exchange. Proteoglycans were also isolated from conditioned media from eS2ED transfected cells using the same methodology. 10 µg of each protein preparation was incorporated into collagen I gels and then seeded with rat aortic rings as described in the Materials and Methods. Sprouts from eight rings were counted and results are mean±s.e.m. ****P<0.0001 (one-way ANOVA comparing to empty vector controls using the Tukey's camparisons test).

We next explored the possibility that the syndecan-2 ectodomain cleaved from the cell surface in response to a known inducer of syndecan shedding, the pro-inflammatory mediator TNFα, could regulate angiogenesis (Pruessmeyer et al., 2010). HEK293t cells transfected with full-length HA-tagged syndecan-2 cDNA (eFLS2, Fig. 2A) expressed an abundance of cell surface syndecan-2 as demonstrated by flow cytometry, and the presence of HS chains was confirmed by western blotting (supplementary material Fig, S3B,C). These cells were stimulated with TNFα for 4 h and the medium was analysed for the HA-tagged shed syndecan-2 ectodomain. TNFα was found to induce shedding of syndecan-2 in a concentration-dependent manner (Fig. 2B). To investigate the potential impact of the shed molecule on angiogenesis, we performed anion exchange on conditioned media to isolate the HSPGs from empty vector and eFLS2-transfected cells stimulated with TNFα, and also on conditioned media from cells constitutively releasing eS2ED. The resulting HSPG-enriched samples were incorporated into collagen matrices into which rat aortic rings were embedded. Rings grown in the presence of empty vector eluates exhibited normal sprout formation. However, eluates containing shed syndecan-2, either TNFα-induced (eFLS2) or constitutively released (eS2ED), strongly inhibited angiogenic sprout formation (Fig. 2C). These data suggest that the anti-angiogenic properties of the syndecan-2 ectodomain are retained when syndecan-2 is shed from the cell surface.

### The anti-angiogenic properties of shed syndecan-2 reside in the extracellular core protein of the molecule

**Fig. 3.**
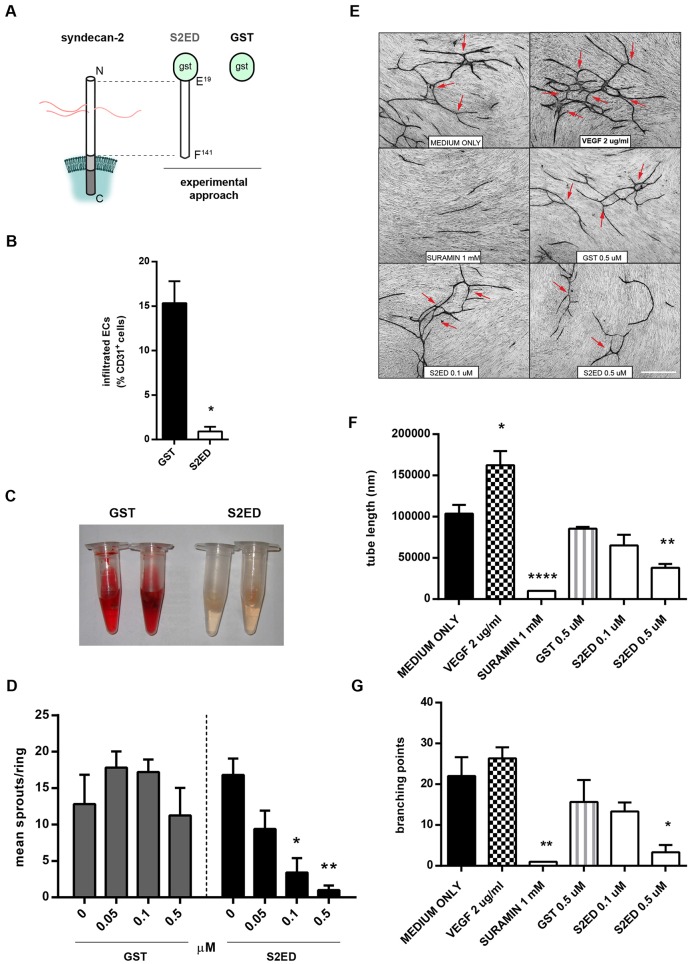
**The syndecan-2 extracellular core protein inhibits angiogenesis.** (A) Schematic diagram of S2ED consisting of GST fused to the syndecan-2 ectodomain core protein. (B) Angiogenesis is inhibited by S2ED when incorporated into Matrigel. C57BL/6 mice were given sub-dermal injections of Matrigel supplemented with VEGF and FGF containing either 0.5 µM of S2ED or GST. Excised sections of Matrigel plugs were immunostained for CD31 (endothelial cells) and DRAQ5 (all cell nuclei) and analysed by confocal microscopy, and the percentage of cells positive for CD31 calculated. Less endothelial cells migrate into Matrigel plugs containing S2ED. Error bars represent the s.e.m. of four separate images. *P<0.05 (Mann–Whitney *t*-test). (C) Photographs of plugs after excision. (D) The anti-angiogenic effects of S2ED are dose dependant. Rat aortic rings were seeded in collagen I containing the indicated concentrations of GST or S2ED and the angiogenic sprouts counted after 4 days. Data were obtained from at least 10 rings from four separate rats per condition. *P<0.05, **P<0.01 (one-way ANOVA comparing to untreated controls using the Tukey's comparisons test). (E) S2ED inhibits tubule formation in a HUVEC co-culture system. Using the V2a kit, HUVECs and fibroblasts were co-cultured in the presence of VEGF, Suramin, S2ED and GST. Micrographs, showing fewer tubules, were obtained in the presence of S2ED and these were shorter (F) and less branched (G; branch points denoted by arrows). Scale bar: 500 µm. Error bars represent the s.e.m. of four separate images. *P<0.05, **P<0.01, ****P<0.0001 (Mann–Whitney *t*-test).

In the following series of experiments, we further explored the impact of the extracellular core protein moiety of shed syndecan-2 on angiogenesis. For this purpose, we used a bacterial expression system to produce a fusion protein consisting of the extracellular core protein [of murine syndecan-2 (E^19^–F^141^) fused at the N-terminus to GST (S2ED, Fig. 3A) (Whiteford et al., 2007; Whiteford et al., 2011)]. The resulting protein is easily purified using glutathione and is not modified with HS chains because prokaryotes lack the requisite transferases (Whiteford et al., 2007). We incorporated S2ED into Matrigel plugs injected sub-dermally in the abdomen of C57BL/6 mice. After 5 days, plugs containing GST alone contained both CD31+ endothelial cells and blood, a possible indication of new blood vessel formation (Fig. 3B,C). In contrast plugs containing S2ED showed a notable reduction in angiogenesis as indicated by a reduced number of CD31+ endothelial cells (Fig. 3B,C). In addition, analysis of Matrigel plugs for haemoglobin content indicated less blood, indicative of reduced vascular formation in plugs incorporating S2ED as compared to those with GST alone (Fig. 3B; supplementary material Fig. S4A).

Having demonstrated the regulatory effects of S2ED on angiogenesis *in vivo*, we expanded these studies to both *ex vivo* and *in vitro* models of angiogenesis. Rat aortic explants were embedded into collagen I gels in which either GST (control) or S2ED was incorporated in the presence of VEGF. Whereas S2ED inhibited sprout formation in a concentration-dependent manner, rings grown in the presence of GST were unaffected by this treatment and sprouted to the same degree as untreated controls (Fig. 3D). S2ED also inhibited VEGF-induced angiogenesis in a model employing aortic rings from C57BL/6 mice (supplementary material Fig. S4B). The effect of S2ED *in vitro* was tested on human umbilical vein endothelial cell (HUVEC) tube formation when in 3D co-culture with human dermal fibroblasts using the commercially available V2A vasculogenesis to angiogenesis kit. After 2 weeks in culture under control conditions, tubule structures were formed with branch points (Fig. 3E). This effect could be augmented with the addition of VEGF and inhibited by the addition of Suramin. The addition of GST to the culture medium had little effect on either the length of tubules formed or the number of branch points as compared to the control medium. In contrast, in the presence of S2ED a significant reduction in tubule length and branch points was noted (Fig. 3F,G). Taken together, these results demonstrate that the syndecan-2 extracellular core protein has anti-angiogenic properties in both rat, murine and human model systems.

### The anti-angiogenic properties of S2ED reside in the syndecan-2 adhesion regulatory domain

**Fig. 4.**
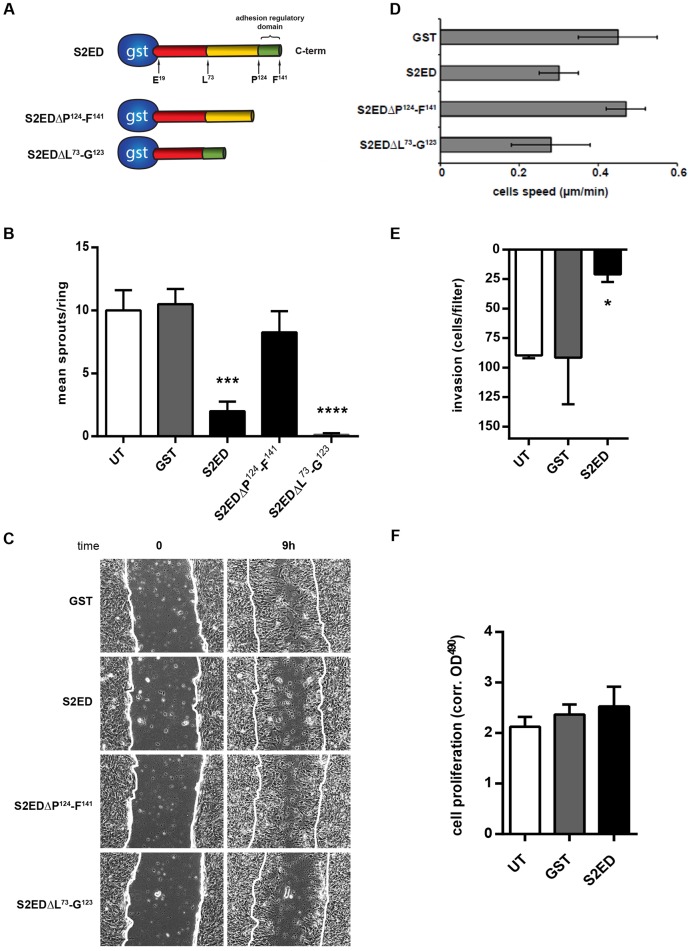
**The anti-angiogenic properties of S2ED are due to inhibition of endothelial cell migration and are mediated by amino acids P^124^–F^141^.** (A) Diagram of the mutant proteins used in this study. Full-length syndecan-2 extracellular core protein S2ED comprising E^19^–F^141^, a truncated form of S2ED lacking the adhesion regulatory domain, S2EDΔP^124^–F^141^ (green), and a mutant containing only the GAG attachment domain and the C-terminal adhesion regulatory domain, S2EDΔL^73^–G^123^. (B) The adhesion regulatory domain of syndecan-2 inhibits angiogenic sprout formation. Rat aortas were seeded in collagen I matrices containing 0.5 µM of GST, S2ED or the mutant forms of S2ED. Sprouts from eight rings per condition were counted and the mean calculated. Error bars represent the s.e.m. ***P<0.005, ****P<0.0001 (one-way ANOVA with Bonferroni multiple comparison). (C,D) Scratch wound migration assays were performed in the presence of 0.5 µM of the indicated fusion proteins on brain endothelial cells. (D) Endothelial cell migration speed is reduced in the presence of S2ED proteins containing the 18-amino-acid regulatory domain. Cell speed data represents mean±s.e.m. measurements from at least 25 individual cells at the leading edge per treatment. (E) S2ED inhibits endothelial cell invasion into collagen I matrices. GST or S2ED (0.5 µM) were incorporated into collagen I matrices in transwells. After 6 h, cells that had migrated were counted and the data represents the mean±s.e.m. of triplicate assays. *P<0.05 (by Student's *t*-test). (F) Cell proliferation is unaffected by S2ED. Confluent monolayers of sEND cells were grown in the presence of 0.5 µM of the proteins indicated and mean proliferation measurements were calculated from four wells per condition.

Given that we have previously shown that fibroblast adhesion to S2ED is regulated by the C-terminal 18-amino-acid domain between P^124^ and F^141^ of murine syndecan-2 (Whiteford et al., 2011), we hypothesised that this adhesion regulatory region of syndecan-2 might also be responsible for the inhibition of angiogenesis. This was initially investigated by performing rat aortic ring assays with deletion mutants of S2ED (Fig. 4A). Full length S2ED, S2EDΔP^124^–F^141^ (lacking the adhesion regulatory domain) or S2EDΔL^73^–G^123^ (a truncated form containing only the adhesion regulatory residues) were incorporated into collagen matrices in which aortic ring sections were embedded (Fig. 4A,B). Although angiogenic sprouts were observed in both untreated and GST controls, sprout formation was severely compromised when rings were embedded in matrices with S2ED or S2EDΔL^73^–G^123^ both of which contain the regulatory 18-amino-acid motif (Fig. 4B). These data indicate that the anti-angiogenic properties of S2ED are dependent on the adhesion regulatory domain lying between P^124^ and F^141^ of murine syndecan-2.

### S2ED inhibits endothelial cell migration

As endothelial cell migration is a crucial component of angiogenesis, the following series of experiments aimed to investigate the effect of S2ED on this response. To establish whether the anti-angiogenic effect of S2ED is due to the inhibition of endothelial cell migration by residues contained within the 18-amino-acid regulatory domain, we performed migration assays on brain endothelial cells in the presence of either S2ED or the truncated forms of this protein (S2EDΔP^124^–F^141^ and S2EDΔL^73^–G^123^). As found with the full-length protein, the truncated fusion protein containing only the adhesion regulatory domain (S2EDΔL^73^–G^123^), inhibited endothelial cell migration (Fig. 4C,D). In contrast, the mutant protein lacking the syndecan-2 adhesion regulatory domain did not affect cell migration, with the wound closure being equivalent to that noted with cells treated with GST alone. The inhibitory effect of S2ED on endothelial cell migration was evident regardless of the vascular bed from which the endothelial cells originated because both skin and lung endothelial cells responded to S2ED and the mutant proteins in a similar manner (supplementary material Fig. S4C). Taken together, these data show that the effect of S2ED on endothelial cell migration is regulated by the C-terminal 18-amino-acid regulatory domain.

We also tested the effects of S2ED on endothelial cell invasion into collagen I gels and observed that cell invasion was greatly inhibited when S2ED was incorporated as compared to controls (Fig. 4E). Of importance, neither protein impacted upon cell proliferation (Fig. 3F). These data are in disagreement with the findings of a previous report (Fears et al., 2006) in which it was suggested that S2ED could promote endothelial cell invasion and stimulate longer microtubules in networks formed when endothelial cells are seeded on Matrigel. In trying to recapitulate this work we observed that S2ED inhibited endothelial cell invasion through Matrigel and had no measurable effect on network formation when sEND cells were seeded on Matrigel (supplementary material Fig. S4D,E). These results also show an inhibitory effect on endothelial cell invasion by S2ED and this is likely to be through inhibition of cell migration.

### The inhibition of angiogenesis by S2ED is driven by CD148, leading to changes in β1 integrin activation

**Fig. 5.**
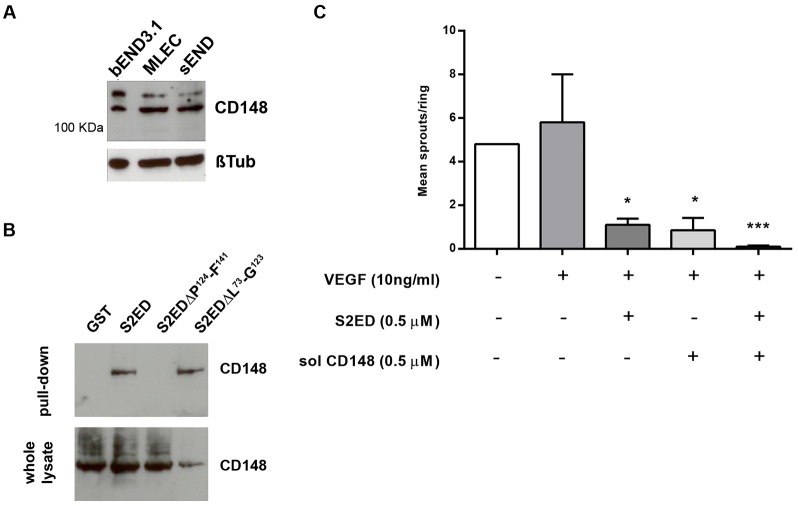
**S2ED interacts with CD148, resulting in the inhibition of angiogenesis.** (A) bEND3.1, MLEC and sEND cells express CD148. Western blot of lysates from the endothelial cell lines indicated were probed with antibodies against CD148 or β-tubulin. (B) CD148 interacts with S2ED. Cell lysates from sEND cells were incubated with beads coated with GST, S2ED, S2EDΔP^124^–F^141^ or S2EDΔL^73^–G^123^. Precipitates were analysed by western blotting for the presence of CD148. CD148 was only pulled down by forms of S2ED which contain the C-terminal 18-amino-acid adhesion regulatory domain. The blot is representative of four experiments. (C) Soluble CD148 inhibits angiogenesis. Rat aortic rings were embedded in collagen I with either 0.5 µM S2ED or 0.5 µM of soluble (sol) CD148, or both proteins in combination, in the presence of VEGF. Both S2ED and soluble CD148 inhibited sprout formation to the same degree, and also inhibited angiogenesis when used in combination. Data are the mean from aortas from five animals and errors bars reflect the s.e.m. *P<0.05, ***P<0.001 (Student's *t*-test comparing values to the untreated VEGF control).

In fibroblasts the protein tyrosine phosphatase receptor CD148 (also known as PTPRJ) interacts directly with the adhesion regulatory domain of S2ED leading to β1-integrin-mediated cell attachment and spreading (Whiteford et al., 2011). To determine whether CD148 is a mediator for endothelial cell responses to S2ED, we first confirmed that endothelial cells from brain, lung and skin expressed CD148. Western blot analysis using an antibody directed to the cytoplasmic domain of CD148 revealed that all three cell lines expressed this receptor (Fig. 5A). We then demonstrated that there was an interaction between CD148 and the syndecan-2 ectodomain by performing a pulldown assay using GST–S2ED beads as bait. No CD148 was evident in precipitates in which GST- or GST–S2EDΔP^124^–F^141^-coated beads were used (Fig. 5B). S2ED and S2EDΔL^73^–G^123^, which both contain the C-terminal 18-amino-acid adhesion regulatory domain successfully pulled down CD148 from endothelial cell lysates indicating that the interaction point between CD148 and S2ED resides in this region of the molecule.

We next performed rat aortic ring experiments using a purified recombinant form of the CD148 extracellular domain comprising the first five type III fibronectin repeats of the human form of this molecule. This also proved inhibitory to angiogenesis to the same degree as S2ED, and when used in conjunction with S2ED the anti-angiogenic effect appeared enhanced (Fig. 5C).

**Fig. 6.**
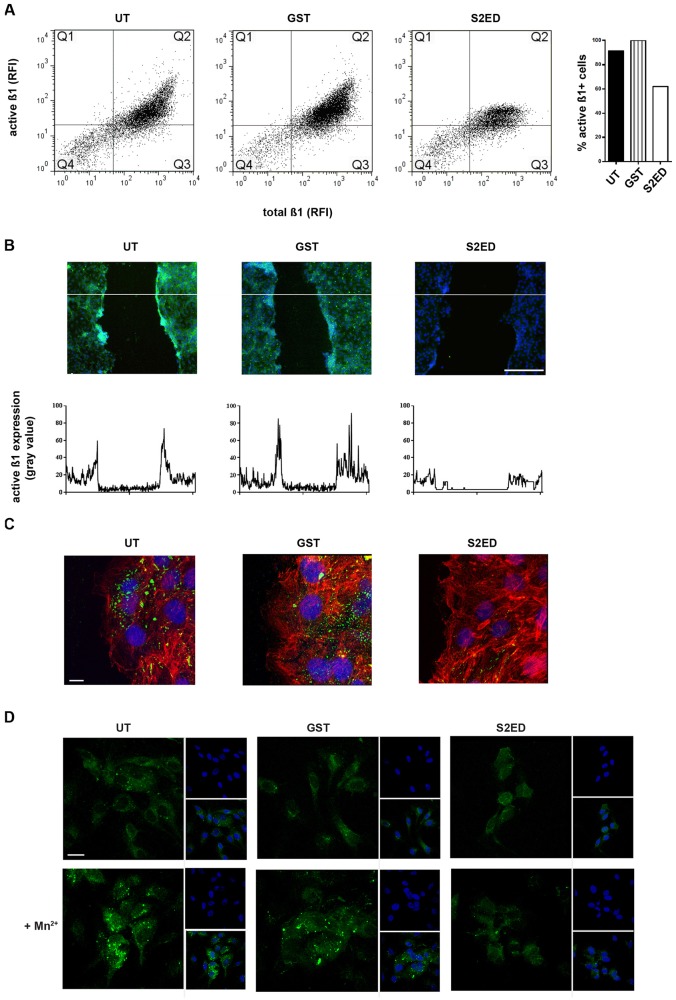
**Active β1 integrin is reduced on endothelial cells in the presence of S2ED.** (A) Active β1 integrin on sEND cells positive for total β1 integrin is reduced in S2ED-treated cells as measured by FACS. The number of endothelial cells with active β1 integrin (as recognised by the monoclonal antibody 9eG7) was expressed as a percentage of total β1-integrin-expressing cells and plotted on the bar chart (right) left and dot blots corresponding to each treatment (left). (B) Micrographs of scratch wounds showing that active β1 integrin is reduced in endothelial cells treated with S2ED. Active β1 integrin is shown in green and DAPI in blue. Scratch wounds were performed on confluent monolayers and were incubated in the presence of the indicated proteins (0.5 µM) for 30 min prior to staining. Graphs represent the fluorescent intensity profile of active β1 integrin across the scratch wounds. The data was obtained from the section represented by the lines indicated on the micrographs. Scale bar: 200 µm. (C) Higher magnification micrographs (63×) of endothelial cells treated as indicated. Scale bar: 10 µm. Cells were stained for F-actin (red), DRAQ5 (blue) and active β1 integrin (green). (D) S2ED causes a reduction in active β1 integrin on HUVECs. HUVECs were treated with 1 mM MnCl_2_ in the presence of 0.5 µM GST or S2ED. Untreated cells or cells treated with GST showed an increase in structures recognised by the monoclonal antibody HUTS4 (green). This was not the case with cells treated with S2ED. Inlays show DAPI staining (blue) and merged images. Scale bar: 20 µm.

The impact of S2ED on endothelial cell β1 integrin activation was examined next. Endothelial cells treated with S2ED showed reduced binding of the β1 integrin activation marker, the monoclonal antibody (mAb) 9EG7, as demonstrated by fluorescence activated cell sorting (FACS) (Fig. 6A). Using this antibody for immunofluorescent staining of endothelial cells, a substantial reduction in active β1 integrins was observed in cells treated with S2ED (Fig. 6B,C). Interestingly this effect was most pronounced at the leading edge of scratch wounds, where the highest expression of active β1 integrins was noted in control and GST-treated cells. We further tested the effect of S2ED on β1 integrin activation in HUVECs. Stimulation of HUVECs with Mn^2+^ lead to an increase in structures that bound the mAb HUTS4 and this was unaffected by the addition of GST. Positive staining was greatly reduced when cells were treated with S2ED (Fig. 6D). Taken together, these results suggest that the anti-migratory effects of S2ED on endothelial cells might be mediated by CD148 signalling, an interaction that could lead to a reduction in active β1 integrins on the surface of endothelial cells (Fig. 6).

## DISCUSSION

**Fig. 7.**
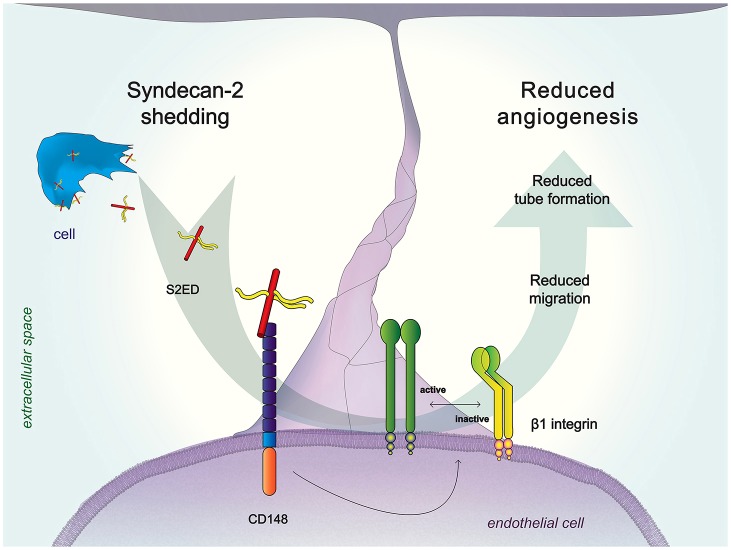
**Engagement of CD148 by shed syndecan-2 corresponds with a reduction in active β1 integrin in endothelial cells leading to inhibition of cell migration and less angiogenesis.**

The use of inhibitors for the suppression of pathological angiogenesis is well established, although clinical evidence suggests that there are a number of complications and side effects (Duda et al., 2007). There is therefore a need for greater understanding of the regulatory mechanisms that both promote and inhibit new blood vessel formation in order to develop new more effective therapeutic targets. We propose a previously unreported role for syndecan-2, in that, in its shed form, syndecan-2 can act to inhibit angiogenesis by slowing endothelial cell migration, through an interaction with CD148 leading to changes in β1 integrin activation (Fig. 7). The identification of the syndecan-2 adhesion regulatory domain as a strong negative regulator of angiogenesis mean that both it and its binding partner CD148 are potential novel therapeutic targets in pathologies where angiogenesis is a feature, such as atherosclerosis, arthritis and cancer.

Angiogenesis requires the action of numerous pro- and anti-angiogenic factors that collectively provide the signals necessary for the generation of new blood vessels. Here, we demonstrate that one such factor is the extracellular core protein of syndecan-2 that, when shed from cells, can negatively regulate angiogenesis. Syndecan shedding is a feature of many cell types and occurs in response to stimuli such as inflammatory mediators and growth factors (Manon-Jensen et al., 2010; Pruessmeyer et al., 2010). Both MMP-7 and MMP-9 have been identified as having a role in the shedding of syndecan-2 (Fears et al., 2006; Kwon et al., 2014). As yet the cleavage sites for syndecan-2 shedding have not been identified; however, the eukaryotic expression system described here will provide an useful tool to address this important question. Shedding studies on syndecan-4, the most closely related member of this family to syndecan-2, suggest that the entire ectodomain can be cleaved from the cell surface (Manon-Jensen et al., 2013). The fact that shed syndecan-2 influences angiogenesis and that the regulatory region resides between P^124^ and F^141^ of the ectodomain, close to the syndecan-2 transmembrane domain, suggests that, like syndecan-4, the entire ectodomain is shed in response to inflammatory stimuli.

Regulatory sequences in the syndecan-1 and, more recently, the syndecan-3 ectodomain have been identified and these have also been shown to be anti-angiogenic (Beauvais et al., 2004; Beauvais et al., 2009; De Rossi and Whiteford, 2013b). However, the evidence presented here suggests that shed syndecan-2 acts on endothelial cells through a mechanism distinct to that associated with syndecan-1 and -3. Syndecan-1 is thought to interact directly with αV integrins, leading to changes in cell adhesion characteristics through an autocrine interaction with IGFR-1 (Beauvais and Rapraeger, 2010; Rapraeger et al., 2013). We propose that shed syndecan-2 acts in a paracrine fashion given that the adhesion regulatory domain of syndecan-2 is situated very close to the transmembrane domain of the full-length molecule. The presence of glycosaminoglycan (GAG) chains and the respective sizes of the receptors involved make it hard to envisage how an autocrine interaction between these CD148 and syndecan-2 could occur. Whether regulation of angiogenesis is a common feature of all four syndecan ectodomains is an attractive possibility that requires further investigation.

We showed that the anti-angiogenic effect of the syndecan-2 ectodomain is observed in both rodent and human models of angiogenesis, and involves the interaction with the protein tyrosine phosphatase receptor CD148 on the surface of endothelial cells, which corresponds with the inactivation of β1 integrins. β1 integrins are intimately associated with angiogenesis and endothelial cell migration (Mettouchi and Meneguzzi, 2006). Our previous work using fibroblasts has shown that engagement of CD148 with S2ED leads to the de-phosphorylation of the p85 subunit of phosphoinositide 3-kinase (PI3K) and that there is a requirement for Src kinase in this process. Furthermore, clustering of CD148 with cognate antibodies leads to β1-integrin-mediated fibroblast adhesion responses (Whiteford et al., 2011). It is likely that this same signalling pathway is functional in endothelial cells, and that these signalling molecules are involved in the reduction in active β1 integrins on the surface of endothelial cells in response to S2ED. CD148 is known to be involved in the inhibition of cell migration in leukocytes, notably neutrophils and macrophages (de la Fuente-García et al., 1998; Zhu et al., 2008; Zhu et al., 2011), and, here for the first time we show it also has a role in regulating endothelial cell migration. Another ECM component, thrombospondin, has also been identified as a ligand for CD148, and this molecule is also known to bind HS, and is an inhibitor of angiogenesis (Takahashi et al., 2012). It is tempting to speculate that these three molecules are involved in some form of cell migration regulatory complex within the ECM, particularly as endothelial cell responses to thrombospondin-1 have been shown to involve β1 integrin.

Syndecan-2 and CD148 are molecules intimately associated with the vasculature. Syndecan-2 is expressed on fibroblasts, leukocytes and endothelial cells, and studies in zebrafish have revealed that syndecan-2 is essential for branching angiogenesis (Chen et al., 2004). Here, we show that CD148 is present on endothelial cells from three distinct vascular beds (brain, skin and lung). CD148-null mice are viable and show no gross defects; however, a knock-in mouse expressing a catalytically dead form of CD148 in which the cytoplasmic domain is replaced by eGFP dies early in development due to a failure in vascularisation (Takahashi et al., 2003; Trapasso et al., 2006). Quite why the cytoplasmically mutated form of CD148 elicits such a strong phenotype is not clear. Our study suggests that engagement of CD148 by its ligands results in an ablation of the angiogenic process and this is supported by the fact that antibodies targeting CD148 have anti-angiogenic affects (Takahashi et al., 2006). We hypothesize that this CD148-driven response to S2ED represents a signal whereby new blood vessel formation is halted, for example at the site of tissue injury. In as much as promoting new blood vessel formation is an important feature of wound healing, the cessation of this process also represents an important step. Syndecan-null animals also breed normally, are fertile and have no gross abnormalities and it is only under challenge or insult that these animals exhibit phenotypes (Ishiguro et al., 2002). It is conceivable that the CD148-null mouse would exhibit similar responses. An intriguing question would be how this mouse would respond to wounding or models where excessive angiogenesis occurs, such as in retinal angiogenesis or tumour models. This work presents an unreported pathway for the inhibition of angiogenesis and presents an alternative therapeutic target for anti-angiogenic therapy to those currently on the market.

## MATERIALS AND METHODS

### Fusion proteins and antibodies

GST, S2ED, S2EDΔP^124^–F^141^, S2EDΔL^73^–G^123^ and soluble CD148 were purified as described previously (Whiteford et al., 2011). Antibodies used were anti-CD148 (R and D Systems), anti-CD29 clone 9EG7 (BD Pharmingen), anti-mouse CD29 clone HMβ1-1 (Biolegend), anti-CD29 clone HUTS4 (Millipore), Anti-HA (clone HA.11, Covance), anti-syndecan-2 (AF6585, R and D systems) and anti β-tubulin (clone TUB2.1, Sigma) antibodies, and Alexa-Fluor-488-conjugated anti-mouse CD31 antibody (Clone MEC 13.3, Biolegend). Alexa-Fluor-568-conjugated phalloidin was from Life Technologies.

### Cell culture

All cells used in this study were grown at 37°C under 10% CO_2_ in Dulbecco's modified Eagle's medium (DMEM; PAA), supplemented with 10% FBS, 2 mM L-glutamine, 1% non-essential amino acids, 1 mM sodium pyruvate and 5 µM β-mercaptoethanol (all Invitrogen). Primary murine lung endothelial cells were isolated and maintained as described previously (Reynolds et al., 2002). HUVECs (HPA laboratories) were grown and maintained in Endothelial Growth Medium (HPA laboratories) and maintained as above.

### Generation of syndecan-2-expressing cell lines

Gene synthesis of the complete murine syndecan-2 cDNA and the cDNA encoding only the syndecan-2 ectodomain coding sequence was performed by GeneArt (Invitrogen). Both full-length and truncated syndecan-2 cDNAs were mutated such that the HA epitope was inserted between D^27^ and K^28^ (supplementary material Fig. S1). *Bam*HI sites were also incorporated at the 5′ and 3′ end of the two synthetic genes. The cDNAs were cloned into the *Bam*HI site of the lentiviral vector pLNT-SFFV-MCS-EGFP (available from T.R.M.). Lentiviruses were produced in HEK293t cells and packaged into a VSVG coat using conventional procedures. HEK293t cells were transfected using the supernatant transfer method. Cells expressing high levels of eGFP were sorted by flow cytometry and these were cultured in DMEM as described.

### Tumour xenograft model

Six-week-old SCID SHO mice (Charles River) were subcutaneously injected in the left flank with 3×10^6^ HEK293t cells stably transfected with either empty vector or eS2ED in 100 µl PBS. Mice were left for 21 days prior to being killed by cervical dislocation. All experiments were performed under the UK legislation for the protection of animals in accordance with UK Home Office regulations. Tumours were dissected and photographed and tumour mass and diameter was measured. Tumours were then snap-frozen in liquid nitrogen prior to embedding in OCT and sectioning (20 µm sections). Sections were fixed using ice-cold methanol and immunostained with anti-CD31 antibodies conjugated to Alexa Fluor 564 and imaged using a PASCAL laser-scanning confocal microscope (Carl Zeiss) with a 10× objective.

### Matrigel plug assay

Male 6-week-old C57BL/6 mice were given subdermal abdominal injections consisting of 600 µl of Matrigel (BD Biosciences) mixed with 100 µl of PBS containing 100 ng/ml VEGF, 100 ng/ml FGF and 20 U/ml of heparin and 0.5 µM of either GST or S2ED (following the method described in Passaniti et al., 1992). Mice were killed after three days and the plugs excised, photographed and incubated overnight in PBS. Haemoglobin was quantified using Drabkin's reagent (Sigma) as described by the manufacturer. All experiments were performed under the UK legislation for the protection of animals, and at the end of all *in vivo* procedures involving anaesthesia, animals were humanely killed by cervical dislocation in accordance with UK Home Office regulations. Excised plugs were frozen in liquid nitrogen and subsequently embedded in ice cold OCT, 15-µm sections were made using a cryostat at −20°C. Following fixation with 4% paraformaldehyde (PFA) for 5 min and blocking with 10% normal goat serum for 15 min, sections were stained for nuclei (DRAQ5) and the endothelial cell marker CD31 overnight. Images were captured using a PASCAL laser-scanning confocal microscope (Carl Zeiss, 10×objective). Quantification was performed using the IMARIS software and Photoshop (Adobe), endothelial cells were identified by co-localization of nuclei with CD31 staining.

### Aortic ring assay

Thoracic aortas dissected from cervically dislocated 180–200 g male wistar rats (Harlan laboratories) or 6-week-old C57BL/6 mice (Charles River), and were sliced into 0.5-mm sections and incubated overnight in serum-free OptiMEM (Invitrogen) at 37°C. Aortic rings were embedded in type I collagen (1 mg/ml) in E4 medium (Invitrogen) containing GST, syndecan-2 mutant proteins or peritoneal exudates in 48-well plates. Wells were supplemented with OptiMEM with 1% FBS and 10 ng/ml VEGF (R and D systems, 30 ng/ml for murine rings) and incubated at 37°C under 10% CO_2_. Angiogenic sprouts from rat and mouse aortas were counted after 4 days and 8 days respectively (method based on that of Nicosia and Ottinetti, 1990 and modified in De Rossi et al., 2013).

### *In vitro* tubule formation assays

HUVECs and human dermal fibroblasts were co-cultured using the Cell Works V2a kit (Caltag Media Systems as described by the manufacturer). Medium containing control compounds, GST or S2ED was changed every second day and at day 14 endothelial cells were stained for CD31 to assess the tube formation. Imaging was performed using an Olympus IX81 inverted microscope (10× objective); tube length and branch points were quantified using Photoshop (Adobe). A branch point was defined as the point at which two or more tubules met, and the tubule length as the length of tubules between branch points. In a second type of microtubule assay, sEND cells (5×10^4^) were seeded into 24-well plates coated with 150 µl of Matrigel (BD sciences) in the presence of either GST or S2ED. Using the Cell-IQ controlled environmental chamber (CM technologies) plates were incubated at 37°C under 10% CO2 and images were captured every 15 min for 16 h.

### Scratch wound migration assays

Scratch wound migration assays were performed on confluent monolayers of endothelial cells. Wounds were made using a pipette tip, and fusion proteins were added to the medium and images were captured every 30 min for 12 h by time lapse microscopy using an Olympus IX81 microscope. Percentage wound closure was calculated and individual cells were tracked using Image J.

### Invasion assay

Invasion assays through collagen and Matrigel matrices were performed in 24-well plates with transwell inserts [Millipore; 8-µm pore size, polyester (PET) membrane]. Membranes were coated with 10 µl of a collagen type I mixture (Millipore; 1 mg/ml in E4 medium) containing 0.5 µM GST or S2ED. sEND cells were seeded on the gel in a homogenous single cell suspension of 5,000 cells/insert in 200 µl of DMEM plus 10% FBS; 1 ml of the same medium was added to the bottom well. Invasion was measured after 6 h after which time gels were removed with a cotton swap, the filter washed in PBS and stained with calcein (Invitrogen) and the number of cells attached to the filter counted.

### Proliferation

Cell proliferation was measured using the CellTiter 96 AQ_ueous_ Cell proliferation assay kit as described by the manufacturer (Promega).

### CD148 pulldown and western blotting

Confluent bEND3.1 cells were lysed in 1% Triton X-100 in Tris-buffered saline (TBS) containing HALT protease and phosphatase inhibitors (Pierce). GST and S2ED were bound to glutathione–Sepharose beads (GE Healthcare) and were added to cell lysates and incubated for 1 h. Beads were isolated by centrifugation and washed twice in TBS, prior to incubation in Laemmli buffer and analysis by western blotting using standard procedures.

### Dot blotting

Samples were diluted in blotting buffer (0.15 M NaCl buffered to pH 4.5 with 50 mM sodium acetate, with 0.1% Triton X-100) and applied under vacuum to cationic nylon membranes (GE Healthcare; Amersham Hybond™-N+). Membranes were washed three times with blotting buffer, blocked for 1 h in blocking buffer (3% milk, 0.5% BSA, 0.15 M NaCl in 10 mM Tris-HCl pH 7.4) and incubated overnight with primary antibody in blocking buffer plus 0.3% Tween-20. After washing, blots were incubated for 2 h with the appropriate horseradish peroxidase (HRP)-conjugated secondary antibody and signals were detected by chemi-luminescence using conventional procedures. Quantification of the signal intensity was performed using ImageJ software.

### FACS and immunofluorescence staining for active integrin

Confluent bEND3.1 cells were trypsinised and the trypsin inactivated with BSA. Cells were re-suspended in Hank's buffer (without Ca^2+^ and Mg^2+^) containing the treatments described (0.5 µM GST or S2ED) and incubated for 30 min at 37°C. Cells were then fixed in 2% PFA prior to FACS analysis for both total and active β1 integrin and the percentage of cells expressing active β1 calculated. For immunofluorescence staining of active β1 integrin, confluent monolayers of sEND cells were grown on microscope slides and scratch wounds were made and either 0.5 µM GST or S2ED was added. After 30 min, cells were fixed with 4% PFA (Sigma) and permeabilised in 0.1% Triton X-100 (Sigma) in PBS. Samples were incubated with active β1-integrin-specific antibody 9EG7 in 1% normal goat serum in PBS overnight at 4°C. Slides were washed in PBS and incubated in anti-rabbit-IgG antibody conjugated to Alexa Fluor 488 (Molecular Probes) and DAPI, and images were captured on an Olympus IX81 inverted microscope. Fluorescent intensity profiles from the resulting images were calculated using ImageJ. For higher resolution images, cells were imaged using a PASCAL laser-scanning confocal microscope (Carl Zeiss) with a 63× objective and the resulting stacks were processed using IMARIS software. HUVECs were seeded on coverslips coated with fibronectin (10 µg/ml) and collagen I (30 µg/m) in 0.1% gelatin. Once ∼60% confluent, cells were washed with serum-free OptiMEM then treated either with or without 1 mM MnCl_2_ in the presence of either 0.5 µM GST or S2ED for 30 min. Cells were then fixed in 2% PFA and stained using a monoclonal active β1-integrin-specific antibody (clone HUTS4, at 1∶300). After washing, cells were stained with an anti-mouse-IgG antibody conjugated to Alexa Fluor 594 (Molecular Probes). Cells were analysed by confocal microscopy as described above.

## Supplementary Material

Supplementary Material
