# Supplementary material for: Shed syndecan-2 inhibits angiogenesis
Source: J Cell Sci. 2014 Nov 1;127(21):4788–99. doi: 10.1242/jcs.153015 (PMC4215719; doi:10.1242/jcs.153015)
Supplement: Supplementary Material [file supp_127_21_4788__index.html]

Shed syndecan-2 inhibits angiogenesis — Supplementary Material 

# Shed syndecan-2 inhibits angiogenesis

## JCS153015 Supplementary Material

**Files in this Data Supplement:**

- **Supplementary Material**
