# Supplementary material for: Shed syndecan-2 inhibits angiogenesis
Source: J Cell Sci. 2014 Nov 1;127(21):4788–99. doi: 10.1242/jcs.153015 (PMC4215719; doi:10.1242/jcs.153015)
Supplement: Supplementary Material [file supp_127.21.4788_JCS153015.pdf]

SUPLEMENTARY MATERIAL

Supplemental Figure 1

```
  1: R  I  L  G  A  T  S  L  G  N  M  Q  R  A  W  I  L  L  T  L
     AGGATCCTAGGAGCCACATCCCTGGGGAATATGCAGCGCGCGTGGATTCTGCTCACCTTG
   1 ---------!---------!---------!---------!---------!---------! 60
     TCCTAGGATCCTCGGTGTAGGGACCCCTTATACGTCGCGCGCACCTAAGACGAGTGGAAC

  1: G  L  M  A  C  V  S  A  E  T  R  T  E  L  T  S  D  Y  P  Y
     GGCTTGATGGCCTGTGTGTCCGCAGAGACGAGAACAGAGCTGACATCCGATTATCCATAT
  61 ---------!---------!---------!---------!---------!---------! 120
     CCGAACTACCGGACACACAGGCGTCTCTGCTCTTGTCTCGACTGTAGGCTAATAGGTATA

HA epitope insertion
  1: D  V  P  D  Y  A  K  D  M  Y  L  D  N  S  S  I  E  E  A  S
     GACGTGCCAGACTATGCTAAGGATATGTACCTTGACAATAGCTCCATTGAGGAAGCTTCA
 121 ---------!---------!---------!---------!---------!---------! 180
     CTGCACGGTCTGATACGATTCCTATACATGGAACTGTTATCGAGGTAACTCCTTCGAAGT

  1: G  V  Y  P  I  D  D  D  D  Y  S  S  A  S  G  S  G  A  D  E
     GGAGTATATCCTATTGATGATGATGACTATTCTTCTGCCTCAGGCTCAGGAGCTGATGAA
 181 ---------!---------!---------!---------!---------!---------! 240
     CCTCATATAGGATAACTACTACTACTGATAAGAAGACGGAGTCCGAGTCCTCGACTACTT

  1: D  I  E  S  P  V  L  T  T  S  Q  L  I  P  R  I  P  L  T  S
     GACATAGAGAGTCCAGTTCTGACAACATCCCAACTGATTCCAAGAATCCCACTCACTAGT
 241 ---------!---------!---------!---------!---------!---------! 300
     CTGTATCTCTCAGGTCAAGACTGTTGTAGGGTTGACTAAGGTTCTTAGGGTGAGTGATCA

  1: A  A  S  P  K  V  E  T  M  T  L  K  T  Q  S  I  T  P  A  Q
     GCTGCTTCCCCCAAAGTGGAAACCATGACGTTGAAGACACAAAGCATTACACCTGCTCAG
 301 ---------!---------!---------!---------!---------!---------! 360
     CGACGAAGGGGGTTTCACCTTTGGTACTGCAACTTCTGTGTTTCGTAATGTGGACGAGTC

  1: T  E  S  P  E  E  T  D  K  E  E  V  D  I  S  E  A  E  E  K
     ACTGAGTCACCTGAAGAAACTGACAAGGAGGAAGTTGACATTTCTGAGGCAGAAGAGAAG
 361 ---------!---------!---------!---------!---------!---------! 420
     TGACTCAGTGGACTTCTTTGACTGTTCCTCCTTCAACTGTAAAGACTCCGTCTTCTCTTC

  1: L  G  P  A  I  K  S  T  D  V  Y  T  E  K  H  S  D  N  L  F  *
     CTGGGCCCTGCTATAAAAAGCACAGATGTGTACACGGAGAAACATTCAGACAATCTGTTTTAA
 421 ---------!---------!---------!---------!---------!---------! 480
     GACCCGGGACGATATTTTTCGTGTCTACACATGTGCCTCTTTGTAAGTCTGTTAGACAAA

  1: K  R  T  E  V  L  A  A  V  I  A  G  G  V  I  G  F  L  F  A
     AAACGGACAGAAGTTCTAGCAGCCGTCATTGCTGGTGGTGTGATCGGCTTTCTCTTTGCC
 481 ---------!---------!---------!---------!---------!---------! 540
     TTTGCCTGTCTTCAAGATCGTCGGCAGTAACGACCACCACACTAGCCGAAAGAGAAACGG

  1: I  F  L  I  L  L  L  V  Y  R  M  R  K  K  D  E  G  S  Y  D
     ATTTTCCTCATCCTGCTATTGGTGTACCGCATGCGGAAGAAAGATGAAGGAAGCTACGAC
 541 ---------!---------!---------!---------!---------!---------! 600
     TAAAAGGAGTAGGACGATAACCACATGGCGTACGCCTTCTTTCTACTTCCTTCGATGCTG

  1: L  G  E  R  K  P  S  S  A  A  Y  Q  K  A  P  T  K  E  F  Y
     CTTGGAGAACGCAAACCATCCAGCGCAGCTTACCAGAAGGCACCCACTAAGGAGTTTTAT
 601 ---------!---------!---------!---------!---------!---------! 660
     GAACCTCTTGCGTTTGGTAGGTCGCGTCGAATGGTCTTCCGTGGGTGATTCCTCAAAATA

  1: A  *  G  S
     GCATAAGGATCC
 661 ---------!-- 672
     CGTATTCCTAGG
```

Murine syndecan-2 cDNA with HA tag insertional mutation (white text, red background). Syndecan coding sequence for eFLS2 is in bold and sequence encoding eS2ED is underlined with the addition of a stop codon (red).

Supplemental Figure 2

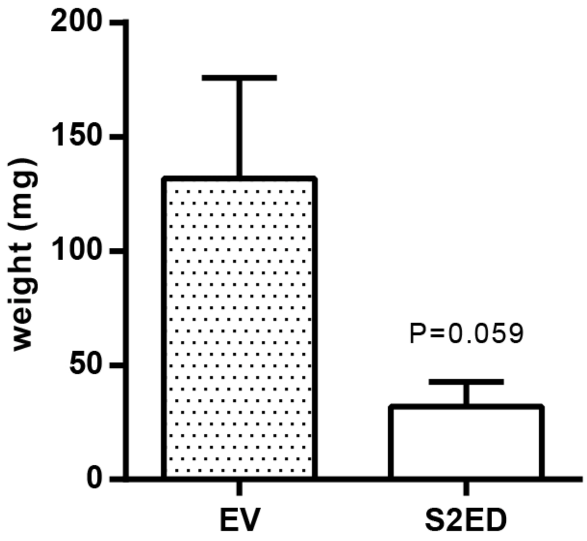

Tumours derived from eS2ED cells have smaller diameter and mass. Error bars represent the SEM and significance was calculated using a Students t test.

Supplemental Figure 3

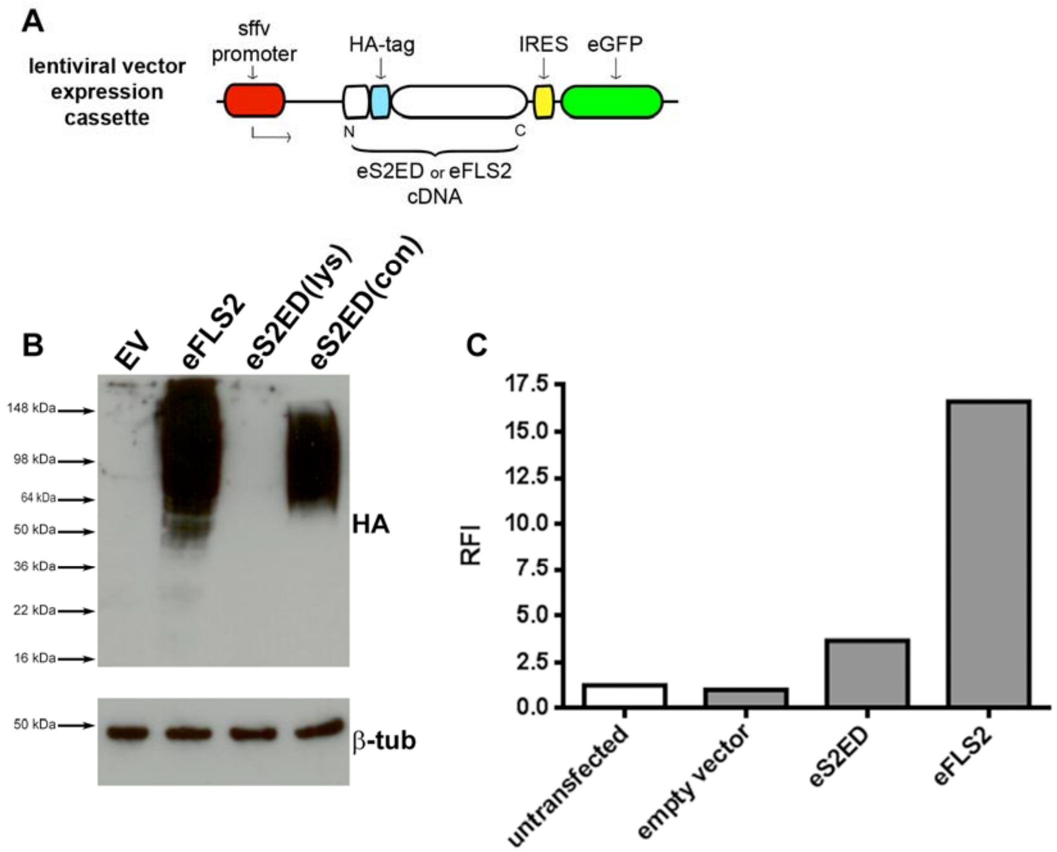

Characterisation of the syndecan-2 expressing HEK293t cell lines. (A) Schematic diagram of the lentiviral expression cassette used for transfection. (B) Expression of full length syndecan-2 was demonstrated by western blot using anti-HA antibodies. A high molecular weight smear is observed in cells expressing eFLS2 indicating that the protein is substituted with HS. No smear or band is evident in lysates from cells transfected with eS2ED (eS2EDlys) however HSPGs isolated from the conditioned media by anion exchange (eS2EDcon) contain a high molecular weight smear indicating that the constitutively secreted syndecan-2 core protein is glycanated. (C) The full length form of syndecan-2 is expressed on the cell surface. Flow cytometry using the anti-HA antibody was performed on the cell lines indicated.

Supplemental Figure 4

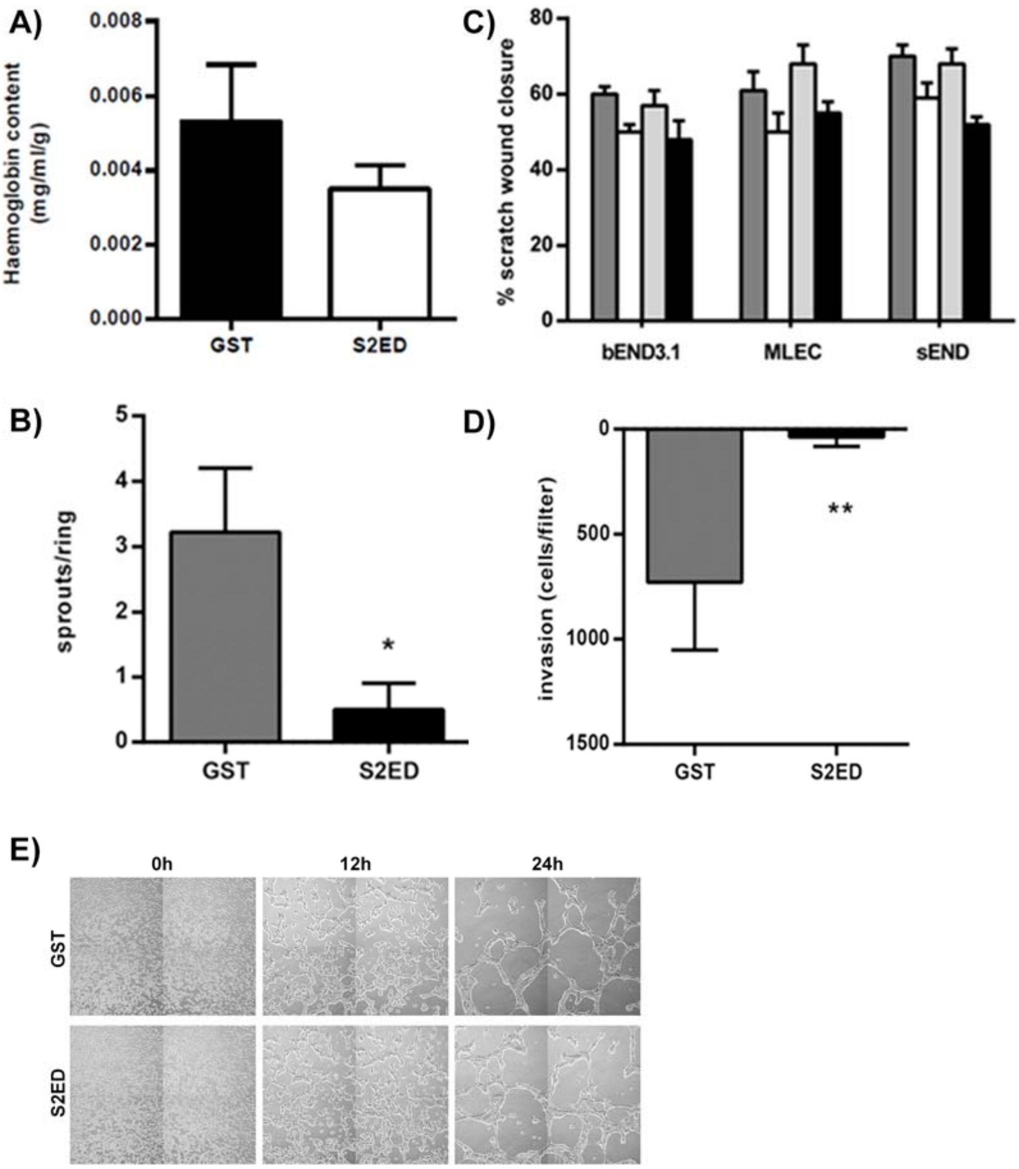

A) Quantification of haemoglobin content of Matrigel plugs. Plugs containing S2ED (white bar) contained less blood than GST control (black bar). Error bars represent the highest and lowest mean measurements obtained from 2 mice injected with 2 plugs.

B) S2ED is inhibitory to angiogenic sprout formation from murine aortas. Murine aortic rings were seeded in collagen I gels containing 0.5μM of GST or S2ED and supplemented with 1% FBS and VEGF. (Statistical analysis was performed using a Student's t-test, n=5; $p<0.05$).

C) S2ED inhibits migration of ECs from different vascular beds. Scratch wound migration assays were performed in the presence of 0.5µM of the fusion proteins indicated on the EC cell lines from brain, lung and skin. Percentage scratch wound closure was measured after 9 hours of incubation. Proteins containing the adhesion regulatory domain S2ED (white bars) and S2EDΔL^{73}-G^{123} (black bars) inhibit EC migration whereas migration is unaffected by treatment with GST (grey bars) and S2EDΔP^{124}-G^{123} (light grey bars).

D) S2ED slows EC migration through Matrigel. S2ED inhibits EC invasion through Matrigel. GST or S2ED (0.5µM) was incorporated into Matrigel layers in transwells. After 6 hours migrated cells were counted and the data represents the mean of triplicate assays.

E) EC microtubule formation is not affected by S2ED. sEND cells were seeded on layers of Matrigel and micrographs were obtained at the times indicated.
